# The Meaning of Mental Imagery in Acute Suicidal Episodes: A Qualitative Exploration of Lived Experiences

**DOI:** 10.1177/00302228231218562

**Published:** 2023-11-25

**Authors:** Anna Maria Nilsson, Margda Waern, Anna Ehnvall, Ingela Skärsäter

**Affiliations:** 1 Department of Psychiatry and Neurochemistry, 195564 Institute of Neuroscience and Physiology, University of Gothenburg, Gothenburg, Sweden; 2 Västra Götalandsregionen, Psychosis Clinic, Sahlgrenska University Hospital, Gothenburg, Sweden; 3 Gothenburg Centre for Person-centred Care, University of Gothenburg, Gothenburg, Sweden; 4 Psychiatric Outpatient Clinic, Varberg, Sweden; 5 School of Health and Welfare, Centre of Research on Welfare, Health and Sport, 3694 Halmstad University, Halmstad, Sweden

**Keywords:** acute suicidal episodes, mental imagery, qualitative content analysis, repeated in-depth interviews, suicidal cognitions interview

## Abstract

Clinical assessment of suicidal ideation focuses on cognitions in the form of verbal thoughts. However, cognitions also take the shape of mental imagery. The aim of this qualitative study was to explore the meaning of mental imagery in acute suicidal episodes (ASEs). Eight persons with severe previous ASEs participated in repeated in-depth interviews and in the semi-structured Suicidal Cognitions Interview. Textual data from both sources underwent content analysis. All participants experienced suicide-related imagery during ASEs. Analysis resulted in two themes. (1) Suicide-approaching imagery: intrusive looming images that contributed to loss of control, flashforwards that clarified the suicidal solution, or desirable but unattainable images. (2) Suicide preventive imagery: death-alienating, life-affirming, or potentially helpful images. The meaning of mental imagery in ASEs is suggested to be understood in relation to the context of the individual ASE. A narrative approach is encouraged, as is an increased clinical focus on mental imagery in general.

Suicide is a major public health concern and a leading cause of death in young people. Globally, more than 700 000 people die of suicide every year and many more attempt suicide (WHO, 2021). Furthermore, there is still a challenging task to assess and treat patients presenting with suicidality in mental health care (Aldrich & Cerel, 2022; Waern et al., 2016). Traditionally, clinical risk assessments address suicidal cognitions in the form of verbal thoughts, although it is well-known that cognitions can take the shape of mental imagery (Beck, 1976). Mental imagery is a broad multi-sensory concept, including anything from fleeting sensory impressions and static scenes to detailed pre- or re-play of events in a specific time and space (Ji et al., 2019). Imagery has a larger impact on emotions than verbal processing (Holmes & Mathews, 2005; Mathews et al., 2013; Slofstra et al., 2018) and a strong influence on future behavior (Pearson et al., 2015). Not surprisingly, mental imagery is considered central in psychopathology and therapeutic change (Hackmann et al., 2011).

Youths who self-injure has been reported thinking in images while the urge to self-injure was strong, and imagery has been suggested to be a potentially important variable underlying non-suicidal self-injury, NSSI (Hasking et al., 2018). When suicidal, a person appears to have realistic and compelling imagery parallel to verbal thoughts (Crane et al., 2011; Hales et al., 2011; Holmes et al., 2007; Ng et al., 2016). Such imagery might be intrusive flashbacks from previous suicide attempts (Chiles & Strosahl, 2004), or “flashforwards” to a future suicide attempt (Holmes et al., 2007). Suicidal imagery is assumed to be associated with high levels of suicidal intent and may facilitate transition from ideation to action (Ji et al., 2019). Conversely, it has been suggested that imagery may reduce the risk of suicidal acts (Crane et al., 2011).

One knowledge gap concerns the absence of a detailed history of occurrences, e.g which makes it impossible to delineate whether imagery was experienced prior to or following suicidal acts. Furthermore, a more comprehensive interview that may reveal multiple suicidal images per participant has been requested (Crane et al., 2011). While most previous work on suicidal imagery is based on quantitative data, one interview study (Hales et al., 2011) applied qualitative analysis. Appraisals of flashforwards involved metacognitive interpretations and negative self-cognitions, and responses included suicidal acts or engaging in distraction or avoidance. Imagery was suggested to be as comforting as it was distressing.

To date, there is insufficient understanding of the role that mental imagery may play in acute suicidal episodes (ASEs). Time-limited ASEs are characterized by activation of interdependent systems (cognitive, affective, behavioral, physiological) heightening suicide risk, and mental images are suggested to function as parts in the cognitive (suicidal belief) system, and/or as internal triggers to the ASEs (Rudd, 2000). As of yet, no qualitative imagery studies involve outlining of the context in ASEs as experienced by the persons. The importance of context and complexity is highlighted in the suicidology debate (Hjelmeland & Knizek, 2010), arguing for qualitative inquiry. By exploring ASEs through qualitative in-depth interviews, the function and significance of imagery for suicidality may be elucidated.

The aim of this study is to gain a deepened understanding of the role of mental imagery, more specifically to explore in detail its meaning in ASEs as well as its potential of being more helpful in the future.

## Method

In addition to the description below, further details of method, recruitment, procedure, and ethics are described in our previous study (Nilsson et al., 2022).

### Participants

Purposeful selection captured heterogeneity (sex, age, diagnosis). Inclusion criteria comprised discernible episodes of acute suicidality, ongoing contact at a mental health service, and ability to speak about own psychological matters. Exclusion criteria included incapacity to participate in interviews due to severe psychosis, substance abuse, dementia, or insufficient language proficiency. Interviews were scheduled to avoid periods of high suicide risk, thus persons with intermittent high risk could participate. Ten persons received study information; nine accepted participation. Imagery data was missing for one who left the study due to an addiction relapse. In all, five women and three men aged 19–66 years were included in the current study (Table 1).

**Table 1.** Participant Characteristics at Baseline (*n* = 8).

Characteristic		n
Age (years)	19–24	2
	25–34	2
	35–45	2
	46–66	2
Living arrangements	Alone	4
	With parents	1
	With wife	1
	With Ex (husband/boyfriend)	2
Highest level of attained education	Highschool	4
	Post-Highschool/University degree	4
Employment	Employed	2
	Sick leave/Retired	6
Main clinical diagnosis	Emotionally unstable personality disorder	3
	ADHD/ADD	2
	Bipolar disorder II	1
	Recurrent depression	2

Participants were recruited from psychiatric outpatient services. Beyond primary diagnoses shown in Table 1, all had a current anxiety disorder, and all were on antidepressant medication. None had a clinically diagnosed alcohol use disorder, but AUDIT indicated risk consumption (Saunders et al., 1993) for seven.

### Design

**Figure 1.**
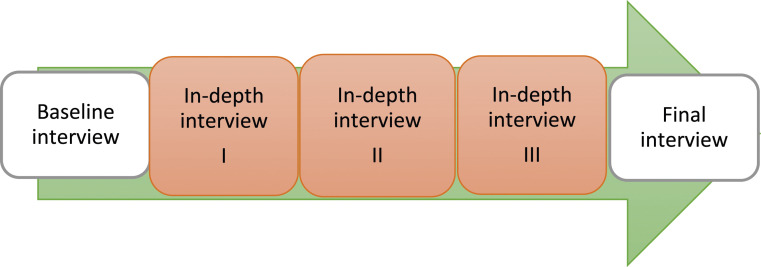
Process chart for interviews.

The project was designed as a series of interviews with each participant (Figure 1).

The first author conducted all interviews. Participants could choose to be interviewed at their outpatient clinic or the hospital research facility. A baseline interview initiated each series to establish contact and collect background data (assessment instruments, medical records). A final interview summarized, highlighted coping strategies, and requested participant validation. Total interview time ranged from eight - 14 hours. The Regional Ethics Review Board in Gothenburg (333-10) approved the study. All participants gave informed written consent.

### Data Collection

#### Scale for Suicide Ideation

The intensity of suicidal ideation and behavior was measured using Beck´s clinician-administered structured scales. The Scale for Suicide Ideation - Current (SSI-C) evaluated at the time of study participation (Beck et al., 1979) and the Scale for Suicide Ideation – Worst (SSI-W) evaluated the time experienced as worst ever in the person´s life (Beck et al., 1999). Higher score indicates greater suicidal preoccupation. Intensity of suicidal ideation at worst point has been shown to associate with future suicide (Beck et al., 1999).

#### Suicidal Cognitions Interview

The semi-structured Suicidal Cognitions Interview (SCI) captures the content of cognitions in the shape of mental images and verbal thoughts (Holmes et al., 2007). SCI is based on the Oxford Guide to Imagery in Cognitive Therapy (Hackmann et al., 2011), according to which assessment of imagery needs to focus on the content, the appraisal of its meaning, the metacognitive understanding of what it means, the impact and response. SCI begins with a definition of the concept of mental imagery. A structured part follows with questions about verbal thoughts and imagery experienced when most suicidal: to estimate the time being preoccupied with suicide-related cognitions, how real and compelling the cognitions felt, and if and how often certain proposed content of cognitions were experienced (nine items, including examples of flashforwards). The last part contains two open-ended prompts:

• ***Describe in as much detail as you can one of the images you experienced when you felt as most despairing or suicidal.*** *Choose the image* that is*/was most important to you. Describe it as if you were a film director and tell as detailed as possible – not just the beginning and the end. Try to include answers to the following questions: What does the image represent? How did it make you feel? What did it mean to you? What did it make you want to do?* Additional questions regard estimation of the distress/comfort associated with the image, how vivid it felt, if it felt as happening now or in the past/future, the perspective from which it was seen (one’s own/outside), and how distressful it felt when imagining it during the interview.
• ***Have you ever experienced any positive, future-oriented images when you have been in a crisis? If so, give a brief description. How could you make this image more helpful to you?***

The interviewer posed the open-ended questions verbally and wrote down the answers verbatim. All but one completed the SCI at the final interview, one did it at the baseline interview. All mental images obtained from SCI were included in the analysis.

#### In-Depth Interviews

Participants underwent three in-depth interviews to elucidate the course of events in ASEs (Nilsson et al., 2022). A pre-developed interview guide posed initial open-ended questions: Can you tell me about the time when you experienced an ASE? What happened during this episode? Describe what happened, from the beginning to the end, as detailed as you can, sequence for sequence. Targeted probe questions followed. Mild ASEs were requested first, thereafter the “worst-ever”. The course of events was reconstructed through collaborative case conceptualization (Kuyken et al., 2008). Each of the three interviews lasted 60–150 minutes, was audio-recorded and transcribed verbatim. The resulting 24 transcripts consisted of 518 pages. The rational for adding this part in the data collection was to put the imagery in its context and penetrate events that surrounded the experience of imaging. Through the initial analysis step described below, 26 descriptions of mental images were identified in the transcripts.

### Analysis

**Figure 2.**
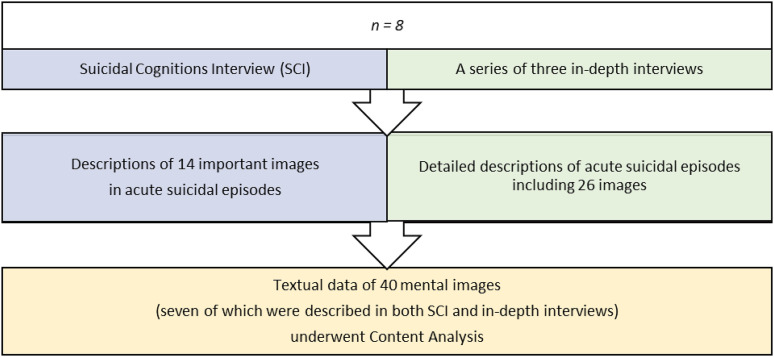
Collection and analysis of qualitative data.

The qualitative data originated from two sources (Figure 2). Resulting data contained detailed descriptions of 40 images as experienced by the participants, including seven pairs of images that were present in both sources.

The analysis emanated from the question - What is the meaning of mental imagery in ASEs? Textual data underwent qualitative content analysis (Baxter, 1991; Graneheim & Lundman, 2004). The author read the texts to get an overall impression. Word constellations referring to experiences of mental imagery in ASEs were identified as meaning units. Condensation reduced meaning units to a core, then marked with labels, codes. Codes must be understood in relation to the text and express the underlying meaning, implying some interpretation. A coding scheme was used (Table 2).

**Table 2.** Coding Scheme With Example From SCI and In-depth interviews (IDI).

Meaning unit (MU)	Condensed MU	Codes	Subtheme	Theme
I think a lot in images… At that time, the image was a clear memory of a previous intoxication with Warfarin. I am alone at home a few days after an overdose of Warfarin, and I am waiting. The image is being expanded. I add that I cut myself in the arm. Blood begins to flow, and I bleed to death, that is a more volatile image… I feel numb. It is as if feelings disappear. Maybe a little calm, no panic, not so scared. I must disconnect to be able to do it… It was the only possible way to have an end to the suffering… It made me practically obtain what is needed to kill myself, made me go to the pharmacy to get tablets, to write down login information, passwords for pages… Then I made a suicide attempt by overdosing (III-SCI)	Memory of a previous suicide attempt: Alone at home waiting after overdose of Warfarin. The image is expanded, I add that I cut my arm and bleed to death … I feel numb. Feelings disappear… I must disconnect to be able to do it… it became the only possible way to end the suffering… I started to practically obtain what is needed to kill myself… went to pharmacy and got tablets. Wrote down login information, passwords… I made a suicide attempt by overdosing	Flashforwards to suicide starting with previous attempt	Flashforwards clarified the suicidal solution	Suicide-approaching imagery
		Images triggered dissociation (dysregulation)		
		Images clarified suicide as solution		
		Images triggered suicidal act		
I see images of myself, and where I would hang myself, myself at home where I hang or where I take the step and how I go about it, if I stand on a chair or something first. Then the image that a next-of-kin or outsider would see, who opens the door and sees me there. Partly the process itself that I do it and then the sight that someone else sees in front of her/him, that I’m hanging in a snare from the ceiling… I have mixed feelings, anxiety, and grief over that I cannot find other solutions, and a little calmness. Both calming and deterrent. I feel resigned… I become tired, that nothing is good. I cannot take it… The suicidal act was hindered (III-IDI)	Concrete images: Partly the process itself, of me trying to kill myself, details of where and how. Partly the sight that another person who arrives would see of me. I Feel anxiety and grief. Images were a little calming but mostly deterrent. I Became tired and resigned, and the suicidal act was hindered	Concrete detailed flashforwards to suicide	Death-alienating images	Suicide preventive imagery
		Images caused fear of death		
		Images had preventive effect		

Co-authors discussed the initial analysis, resulting in modifications of codes. The codes were analyzed and clustered into subthemes. The results were discussed until reaching agreement. From subthemes two themes were developed, expressing the most important latent content.

## Results

All participants had a history of suicide attempts and scored high on SSI-W, median 32 (Table 3), indicating that ASEs were of high degree of severity.

**Table 3.** Characteristics of suicidal Behavior.

Participant (age)	Nr of suicide attempts at baseline	Age at first suicide attempt	Age at worst ASE^a^	Method used in worst ASE^a^	SSI-W score (max 38)	SSI-C^c^ score (max 38)
I (27)	>10	4	25	Poisoning by prescription medication	25	0
II (66)	1	65	65	Poisoning by prescription medication	32	0
III (32)	2	20	27	Poisoning by prescription medication	36	1
IV (66)	5	41	41	Gassing by car in garage	32	18
V (41)	2	21	40	Poisoning by prescription medication	32	10
VI (40)	5	8	38	Cutting	33	5
VII (23)	5	12	23	Jump from balcony	34	15
VIII (19)	9	15	18	Cutting	31	3

^a^Acute suicidal episode.

^b^Scale for suicide ideation-worst.

^c^Scale for suicide ideation current.

According to data from the structured part of SCI (supplementary tables), all participants experienced high preoccupation with detailed mental imagery beyond suicidal verbal thoughts during their ASEs. While the feeling of reality was related as strong in both verbal cognitions and imagery, the latter seemed particularly compelling. Images tended to be concurrently painful and calming. The most frequent content involved images of things one was escaping from. Flashforwards were recognized such as images of oneself preparing to self-harm, or of what would happen to others if one died.

### Meaning of Mental Imagery in ASEs

The qualitative data from the open-ended SCI questions and the deep interviews provided a broad repertoire of mental imagery, including intrusive traumatic negative images, flashforwards to suicide, and positive images of a close person. The imagery appeared suddenly and rapidly, as a single image or as repeated images, a film. Not only visual but also other sensations could be involved, for example auditory. The images tended to be vivid, as if it happened now. The perspective could differ, observing oneself in the image from one’s own perspective, or from the outside. The images might be habitually experienced episodically over years, or snapshots created in the moment.

Our main findings, illustrated in the two themes, Suicide-approaching imagery, and Suicide preventive imagery, showed that the associated meaning of images was individual and situation specific. Regardless of the positively or negatively valued content of the images, their effects might be suicide-approaching or suicide preventive, depending on the individual meaning assigned by the participant. All but one described both suicide-approaching and suicide preventive images, illustrating an ambivalence between life and death. Images appeared as part of the cognitive process, influencing emotions, prior to a potential suicidal act. Hence, the overall function or impact of imagery in ASEs could be facilitative or inhibitive with regard to suicidal behavior. Results are summarized in Table 4 and below in relation to themes and subthemes.

**Table 4.** Overview of themes and Subthemes.

Theme	Subtheme
Suicide-approaching imagery	Looming images
	Flashforwards clarified the suicidal solution
	Desirable but unattainable images
Suicide preventive imagery	Death-alienating images
	Life-affirming images
	Potentially helpful images

Participants’ appraisals of the meaning, metacognitive understanding of the significance, and behavioral response follow.

### Suicide-Approaching Imagery

This theme illustrates images’ function in the ASEs by approaching suicide, elaborated in three subthemes. Imagery could influence the process leading up to a suicidal act. However, whether or not an attempt became fully implemented seemed to be affected by various external or internal conditions (Nilsson et al., 2022).

#### Looming Images

This subtheme addresses the importance of intrusive threatening images that approach at accelerating speed. This is in line with the concept of looming or tendencies to perceive mental simulations of threats as rapidly growing, approaching and rising in risk (Riskind, 1997; Riskind & Rector, 2018). Looming images appeared to contribute to physiological autonomic arousal, ending up in an overwhelmed unbearable state, marked by cognitive short-circuit, where loss of control might happen. Content tended to be traumatic, e.g. previous events, or fantasies built on events that one had heard of, relived repeatedly during the ASEs. Painful emotions and negative selective perception seemed to predominate, with negative views of oneself, others, and the future. The meaning of the images could involve a confirmation of being excluded. Catastrophic generalizing cognitions could escalate before ideation switched to behavior:

“My whole life comes… like a fast movie... It comes like a flash in one second. All the people I have disappointed and who have failed me. I see everything negative… no light… pain within me, in my soul, that much pain I could hardly breath or talk… A lot of anxiety… shame... It’s a lot of pain… in the image… I can’t control anything, not even my own thoughts. The thoughts control me... I’m totally powerless” (VI)

Never-ending difficulties might be experienced metaphorically or symbolically, as in this example before an intoxication:

“I saw a black tunnel… a perception of the future… dark all the way. I just saw misery in front of me. I had painted myself into a corner… I felt I was inside… a ´bubble´ … and got nowhere. (II)

Looming imagery might be in an auditory form, e.g. one’s own voice suggesting to cut oneself, elicited the ASE:

“All of a sudden it came like a voice, what if I would… draw (the knife). It is my own voice like the other times, thoughts that I cannot control… I felt… powerless because it comes as from nowhere. I just want to put an end to it... Panic… ashamed… It pressed against my chest and felt as if my whole thorax shrunk… I felt like a broken item that just as easily can be scrapped” (VII)

Hence, looming images were seen as suicide-approaching, potentially influencing the process leading to suicidal acts.

#### Flashforwards Clarified the Suicidal Solution

This subtheme depicts how imagery enhances the appraisal of suicide as the only remaining solution. Showing the only possible way to end the suffering, the images brought a sense of calm.

“What I saw before me, or thought would happen, was that I would have peace and comfort… Now I know. Now, it will be good. Now I will do it (intoxication with sedatives)” (V)

Flashforwards of what happens after a suicide attempt could be blurry and vague. It might involve dissociative experiences and start with a memory of a previous attempt, e.g. alone at home waiting after an overdose of anticoagulants:

“The image is… expanded. I add that I cut my… arm. Blood begins to flow… I bleed to death… I was numb. Feelings disappear… I must disconnect to be able to do it… The only possible way to have an end to the suffering… It (the image) made me want to get what I would need (pills) to kill myself” (III)

Seeing detailed images of suicidal methods and potential obstacles, could clarify which method might be most appropriate, however this conclusion was made in a state of anxiety and cognitive insufficiency.

“I had very much… anxiety… I needed to calm down… I wanted to do it as mildly as possible for everyone. I did not want to affect my family with having the sight of me hanging at home. I did not want to affect the tram driver’s mind the rest of his life… I started to cut myself” (VIII)

Upon realizing how to proceed the suicidal act seemed to approach closer. Hence, flashforwards with visualization of suicidal methods were indicated as important in suicide-approaching imagery.

#### Desirable but Unattainable Images

This subtheme captures how images with a desirable content can be suicide-approaching if the content is construed as inaccessible. The effect of an image denoted “positive”, could thus be negative. An initial calm and comfort appeared transient when the image was interpreted as far from reality. The feeling might transform into sadness before an intoxication:

“I have a single volatile dream image where I observe myself entirely from the outside. What if it could be like this? I see the image of myself. It is summer. I live in a house with garden and flowers. Someone is with me… My relation to food is… uncomplicated in the image… Then I feel sad… It feels so remote.” (III)

Thus, when positive mental images appear inaccessible, a person might come closer to suicidal action.

### Suicide-Preventive Imagery

This theme describes appraisals of the meaning of images as suicide preventive, illustrated in three subthemes. Imagery could interact in the cognitive process early in the ASEs, resulting in a deterrent and a barrier to proceed to the suicidal act. Moreover, images might function as emotion regulators interrupting initiated attempts. The latter were identified by the participants as helpful strategies with potential to be further developed in the future.

#### Death-Alienating Images

This subtheme depicts how imagery can be suicide preventive by causing fear of death or providing arguments against suicide. Concrete flashforwards appeared, involving details of where and how to attempt suicide, what to use, what obstacles there might be, and what the result would be. Flashforwards could hinder the act:

“I see the process in images in front of me, … myself climbing up the crane… It is dark, cold, and windy. I imagine how it is for real… I get dizzy… I imagine what it looks like and how it feels physically and the actual doing. Then I imagine emotionally, how it will feel to stand there and make the leap. Then what I will look like when I have landed in the asphalt… What is it that I leave behind, that someone will sweep up? I become total mash, lying on the ground with cracked head… I just want to have an end to my suffering. I don’t want to expose those I like… to something… difficult.” (III)

Detailed images of what happens after death appeared. Images might be externally triggered, e.g. an image from a TV documentary (of someone at similar age who died by suicide) was stuck in the mind and developed in fantasy:

“It made me afraid… It meant it could have been me and it became very concrete. I do not want to end up in a place like that... That´s awful. I will die in a way that no one can find me. But it is difficult to make sure it will be done, if I am dead I do not know… Right now, I do not want to die. I do not feel like dying” (I)

The images seemed to concretize what happens after suicide and clarify that the course of events after death are uncontrollable. A death wish could dissipate by realizing the difficulties of implementing a suicidal act, or that no appropriate suicide method exists. One unpleasant death-alienating image showed the Grim Reaper hanging over like a threat, initially paralyzing:

“I was afraid… I had insight into the seriousness of death… and motivation to live. The image made me try to do something to feel better… I gave up the suicide attempt… I need to focus in order to be human again, I concentrated on breathing and how I moved, tried to feel normal… I called the health services instead of swallowing a lot of pills.” (VII)

The fear of death induced by this intrusive image motivated help-seeking. Thus, death-alienating images might in different ways have a suicide-preventive function.

#### Life-Affirming Images

This subtheme illustrates how desirable images affirm the meaning of life and provide a suicide preventive function. Images concerning strength, goodness, change, or connection tended to bring peace, comfort, and instill a sense of freedom. Positive dream images of oneself that appeared could have been created many years ago as a strategy to distract in distressful situations. Mental images of the faces of close persons or pets appeared important. After initiation of a suicidal act, the sudden appearance of a life-affirming image could inhibit completion of the act:

“First it is as if I do not really see that the cats exist, there is nothing. It takes a while and then suddenly it becomes like I really see the cats and feel them. A penny dropped: Suddenly I realize and note, I live for you. I have something to live for. Now I do not want to die anymore. It was interrupted. I stopped taking the tablets.” (I)

Imagery could help regaining access to problem-solving. Abruptly appearing images might function as a cognitive reset, realizing the reasons to live, e.g. reminded of the existence of one’s closest beings. Thus, life-affirming images as reminders of reasons for living could eliminate the need for death and thus have a suicide preventive function.

#### Potentially Helpful Images

This subtheme represents participants’ reasoning and reflections during the interview of how the helpful potential of their images might be enhanced. The common denominators were to make images concrete, real, and accessible. Images of oneself having reached lofty goals in life, e.g. become a professor, the strongest bodybuilder, or the one who saved the world, may be more helpful if the content is toned down and more realistic. Thus, one might be strong, highly respected, and make an impact in a less extreme manner. Other images that were reasoned to have the potential to be helpful involved taking own actions and making a change.

“I saw volatile images… I do something drastically, divorce and move, run away to Tibet and meditate, become a monk, travel, change the environment, meet new people, socialize. It is positive, instills a sense of freedom… come to something good. This could be more helpful if it involved concrete plans, such as plans for the next trip.” (II)

Potentially helpful could mean concretizing something that was available or practically possible. An abstract image of oneself in the summer, together with others, living an uncomplicated life in a house with a flower garden might be transformed:

“I might make the image more concrete… Include a place and persons I already have access to, such as the allotment I share with close friends. Not a fantastic image, but real.” (III)

Positive images seemed helpful if adapted to the possibilities of the moment or modified by adding elements such as moving to be physically closer to persons who they hold dear. Imagery might function as soothing tools in different ways; images of things that could hinder a suicidal act, or prepared reminders to visualize good things available when needed. Seeing the face of a beloved was suggested to function better as a brake or a tool to soothe if the image was adjusted to be more real. Thus, the suicide preventive effect of desirable imagery is proposed to have potential to be enhanced if one makes the images concrete, realistic, and accessible.

## Discussion

### Findings

This qualitative study illuminates the potential importance of mental imagery during ASEs in the themes Suicide-approaching imagery and Suicide-preventive imagery. It is to our knowledge the first to use repeated in-depth interviews to examine images experienced by persons with severe suicidality. Emotionally charged images appeared to influence motivation, planning, and self-regulatory behavior. The appraisals of the images appeared individual with diverse functions and meanings. Furthermore, appraisals tended to be situation-specific, which could be elucidated due to the collection of multiple images per participant. The rich data with details of the course of events in the ASEs provided a deep understanding of the meaning of the imagery in the whole context. This overall picture helped to identify the function and timing of specific images in relation to the whole scenery. Responses to the flashforwards included implementation of suicidal acts.

The theme Suicide-approaching imagery relates to previous findings, by which suicidal imagery was suggested to indicate severe suicidal ideation and to facilitate transition from entrapment to suicidal ideation (Crane et al., 2011; Ng et al., 2016), hence playing part in the motivational phase according to the integrated motivational-volitional, IMV, model (O’Connor, 2011). Imagery has also been suggested as a key factor in the volitional phase of IMV model, when suicidal ideation transforms into action (Hales et al., 2011; Wetherall et al., 2018). In our results this was highlighted by Suicide-approaching flashforwards that seemed to catalyze the switch from ideation to action. According to the interpersonal psychological theory of suicide (Joiner, 2007), habituation to repeated experience of pain is one way of developing a capability of suicide. Habituation may occur by imagery since it resembles actual experiences.

All but one of our study participants related both suicide-approaching and suicide preventive images in their ASEs. We could find no reports in the literature involving both types of mental imagery in persons with previous suicidal behavior and severe worst-point suicidality. Our results contrast with the findings of Crane and colleagues who reported that suicide-preventive imagery only in persons without previous suicidal behavior (Crane et al., 2011). However, it must be stressed that ours is a small qualitative study and as in all qualitative research, results cannot be generalized, and findings should be interpreted cautiously. Nevertheless, our study provides insights of suicidal imagery grounded in personal experiences that might be tentatively put forward to inform future research and clinical practice.

Findings indicate that participants experienced images as distressful and comforting when suicidal, which might represent ambivalence about suicide, in line with previous findings (Hales et al., 2011). However, in partial contrast with other findings which suggest that the experience of imagery as comforting is linked to a history of severe suicidal behavior (Crane et al., 2011), our findings suggests that even persons with severe worst-point suicidality might experience ambivalence reflected in the cognitions, verbal thoughts as well as mental imagery.

Among the images indicated to function as suicide-approaching were looming images. In our previous explorative study of ASEs, looming appeared connected to development of cognitive insufficiency, followed by loss of control and an exceptional state in which suicidal acts might be implemented (Nilsson et al., 2022).

Notably, suicidal flashforwards appeared in both Suicide-approaching imagery and Suicide preventive imagery. Various suicidal methods were considered including the question of how to cause minimal damage to others, but with the result of two opposite functions. The meaning of the suicide-approaching flashforwards seemed to help clarify the suicidal solution. However, this “insight” on how to proceed seemed to occur in a state of cognitive insufficiency, previously described in participants’ experiences of the ASE (Nilsson et al., 2022). Hence the clarification of the suicidal solution was probably not so logically made. The meaning of the suicide preventive flashforwards appeared to increase distance to death through enhanced fear of death or reinforced arguments against suicide. The suicide preventive flashforwards tended to involve more concrete images of suicidal methods including rich details of where and how to do it, what to use, what obstacles there might be, and what the result would be.

Illustrated in “life-affirming images”, images ideally appeared to bring peace, comfort, and instill a sense of freedom, implying an emotional regulation strategy. However, most images experienced during the ASEs, including intrusive looming images, tended to come to mind passively unbidden. Nevertheless, “potentially helpful images”, explored imagery as prospective emotion regulators, illustrating how images actively generated might be comforting.

### Methodological Considerations

A qualitative methodology was chosen because suicidal thinking is a complex phenomenon and there is a need to know more about the subjective experiences and contexts – things that cannot be quantified. With qualitative interviews it is possible to study the experiences in depth and find more nuances in meaning. It is possible to elucidate the suicidal person’s own personal description and provide a wider understanding of the suicidal cognitions than through quantitative methods. (Kjolseth et al., 2009). Preconceptions, previous experiences, and theoretical frameworks always influence the research process. The authors’ background influenced the choice of research questions, methods, and the interpretation of data. The analysis of the result is affected by our previous knowledge and theories even if our aim is to let the data speak for itself. Qualitative content analysis is useful when the purpose of the method is to gain information from study participants, without imposing preconceived categories or theoretical perspectives but based on participants’ unique perspectives and grounded in the actual data (Hsieh & Shannon, 2005).

Several considerations sought to enable trustworthiness, as follows below.

#### Credibility

Data from SCI was enriched with data from in-depth interviews. The meticulous dual data collection brought out complexity and increased trustworthiness. Persons with previous severe suicidal episodes were interviewed with SCI yielding descriptions of significant images from ASEs. Through repeated in-depth interviews about the course of events in ASEs, we obtained individual narratives providing rich material on context. Participant characteristics and background were examined in detail (Nilsson et al., 2022). The methods enabled rich data to be elicited. The participants could share images that gave them a deepened understanding. Choosing participants with various experiences or perspectives, (e.g., genders, ages, diagnostic and social parameters), helped to shed light on the meaning of imagery from a variety of aspects and contributed to rich variation. The selected meaning units, i.e. mental imagery in ASEs, seemed adequately narrow. The inclusion criterion, requiring ability to speak about own psychological matters, influenced the result by prompting clear descriptions. Themes appeared to cover data. Representative quotations were given for every subtheme. Furthermore, credibility was ensured by the inclusion of all authors in the analysis. Co-authors agreed on the final categorization of data. Due to the dual sources of imagery data, and the inclusion of responses to the last questions in SCI (how to make positive future-oriented images helpful), we obtained a broad repertoire of images. Both sources provided detailed data on suicidal cognitions. However, more information, particularly the complexity was brought out by the narrative person-centered approach including the repeated in-depth interviews with each participant, where description and analyses of ASEs were jointly made (Nilsson et al., 2022).

#### Dependability

Data collection is influenced by the knowledge level, experiences, biases, and perspectives of the researcher as well as by what information the participants are willing to or able to provide and what sources of data are available to the researcher (Krippendorf, 1980). Our data is extensive, and the collection extended over time. Hence, alterations made in decisions during the analysis might be an issue. The interview guide ensured that the same areas were covered for all participants. Nevertheless, interviewing was an evolving process during which acquired new insights subsequently influenced follow-up questions or narrowed focus. Inconsistency was dealt with by open dialogue within the research group. Interview data, especially the semi-structured SCI, might involve leading questions. Recall bias is an issue. SCI was completed after the in-depth interviews in all but one participant. Hence, findings from in-depth interviews appeared spontaneously, without influence of SCI questions.

Pre-understanding, built on clinical psychiatric experiences including cognitive behavior therapy, may have influenced the analysis. The theoretical frame of reference consisted of the understanding of suicidality from a biopsychosocial perspective, the concept of ASEs (Rudd, 2000), and the concept of imagery used in cognitive therapy (Hackmann et al., 2011), beyond the understanding of ASEs gained through our previous study (Nilsson et al., 2022). The analysis method included interpretation. Thus, the authors’ pre-understandings certainly influenced the collection and analysis of data, in line with the concept reflexivity (Malterud, 2001), meaning that the perspective or position of the researcher always shapes the research.

#### Transferability

Results from qualitative studies cannot be generalized statistically as the number of participants is limited and the sample is not representative. However, there are other types of generalization. One such is analytical generalization (Kvale & Brinkmann, 2009, Leung, 2015). As our study is set in a Swedish psychiatric outpatient context it is up to the reader to decide the degree to which our findings may be applicable to their own situation. Transferability depends on a well-described strategy for sampling and a well-described context, so that the reader then can appraise to which extent it can be transferred to other groups and situations. To facilitate transferability participants in our study were carefully characterized. It is valuable to give a clear description of selection and characteristics of participants, data collection and process of analysis. A rich and rigorous presentation of the findings together with appropriate quotations will also enhance transferability (Graneheim & Lundman, 2004). We chose a purposeful sample that would bring rich data and meaningful information about the suicidal cognitions - including both men and women, different age-groups, and diagnoses – capturing the heterogeneity among people who suffer from ASEs. Considering the purposeful sampling, our results support the indications of imagery as transdiagnostic with shared characteristics across disorders (Shultebraucks et al., 2020).

Ability to speak about own psychological matters was a requirement for participation, and findings may not be transferable to all clinical situations. Participants with recurrent active suicidality required measures for support post-interview. Three participants had ongoing psychotherapy, the remaining five had supportive contacts (nurse/counselor) at an outpatient clinic. Previous studies concerned participants treated with Mindfulnessbased Cognitive Therapy (Holmes et al., 2007), psychotherapy or CBT (Crane et al., 2011).

To our knowledge, this qualitative study is the first to employ repeated interviews to explore the meaning of mental imagery in ASEs in persons who had experienced severe suicidality. SSI-W scores were far higher than those reported in previous imagery studies. Results may not be transferable to groups without previous severe suicidality/suicide attempts. Persons with a history of suicide attempts tend to be more preoccupied with suicide-related imagery compared to those with suicidal ideation only, or those without suicidal history (Crane et al., 2011).

### Implications

The associated meaning of images appeared individual and situation-specific, which points to the importance of gaining a personal description. Both SCI and the in-depth interviews included open questions posed during a trustful conversation that provided a space to answer freely. Regarding the method of asking, specific questions regarding images might not be necessary as previously suggested (Shultebraucks et al., 2020). A narrative approach with elements of the five-factor model (Padesky, 1990) might enable images to be elicited if important in the context. Thus, reliance, and the detailed analysis of ASEs, seem important in eliciting significant images. As with verbal suicidal cognitions, examining the content of imagery appears insufficient to learn about its impact on behavior in ASEs. We probably need the personal story of the specific appraisal and experienced meaning of each cognition in a specific situation. Even persons considered to suffer from severe suicidality and who have made suicide attempts might present an ambivalence in their ASEs, reflected in both verbal thoughts and mental imagery. This suggests a potential space for intervention. Suicide preventive images might be reinforced into skills for dealing with problems, as suggested in a recent pilot study involving university students (Knagg et al., 2022). Clinicians could be encouraged to a trustful dialogue with the individual, not only as this might reduce future suicidality (Huggett et al., 2022), but also as it might provide the precondition for a detailed narrative, including disclosure of the unique meaning of mental imagery. Furthermore, clinicians could be encouraged to increase attention to mental imagery, penetrate its content, and apply this information in both clinical assessment and treatment. Focusing on the images that emerge might render an elaborated understanding of the persons’ inner suicidal experience. Moreover, the detailed narratives appeared to depict an interaction of external environmental conditions, resulting in actual or interrupted suicide attempts (Nilsson et al., 2022). Thus, beyond the importance of suicidal cognitions, the contextual availability of suicide methods and barriers is suggested essential to consider for a holistic understanding. This is a small qualitative study which implies that the result cannot be generalized. Our findings need to be verified by future quantitative studies with a larger sample. Specific target groups could be explored, as for example individuals with suicidal ideation and those who have attempted suicide respectively, perhaps in an inpatient setting and with ASEs close in time. Quantitative data about imagery could be gained by administration of questionnaires asking direct questions about the meaning of imagery. This approach has several difficulties, such as formulation of relevant questions, if researcher-administered it can be costly, time-consuming, and difficult to analyze. Standardized answers may make it easier to compile data, but participants may find questions irrelevant or difficult to answer, and the design and layout of the questionnaire may not be appealing. They lack detail, because the responses are fixed, there is less scope for respondents to supply answers which reflect their true feelings. However, although there are some difficulties in conducting a larger quantitative study, it could be feasible with the help of data from the qualitative findings.

## Conclusion

Our findings contribute to the development of a deepened understanding of the role of mental imagery as one important component among others in ASEs. The function of imagery appeared suicide-approaching or suicide preventive. Even persons with severe suicidality might have preventive images during their ASEs. The suicide preventive effect is suggested to have the potential to be enhanced if images are modified. To understand the personal meaning of imagery, it seems essential to consider the whole situation in which it appears. The meaning of mental imagery is suggested to be understood in relation to the personal story of ASEs and a narrative approach is thus encouraged when assessing and managing suicidality. Nevertheless, our main findings need to be further verified by quantitative studies. The concepts suicide approaching and suicide preventing imagery need to be tested in a larger, more diverse sample of people with suicidality.

## Supplemental Material

Supplemental Material - The Meaning of Mental Imagery in Acute Suicidal Episodes: A Qualitative Exploration of Lived Experiences Supplemental Material for The Meaning of Mental Imagery in Acute Suicidal Episodes: A Qualitative Exploration of Lived Experiences by Anna Maria Nilsson, Margda Waern, Anna Ehnvall, and Ingela Skärsäter in OMEGA - Journal of Death and Dying
