# Supplementary material for: The Meaning of Mental Imagery in Acute Suicidal Episodes: A Qualitative Exploration of Lived Experiences
Source: Omega (Westport). 2023 Nov 25;92(4):1989–2011. doi: 10.1177/00302228231218562 (PMC12891247; doi:10.1177/00302228231218562)
Supplement: Supplemental Material - The Meaning of Mental Imagery in Acute Suicidal Episodes: A Qualitative Exploration of Lived Experiences [file sj-pdf-1-ome-10.1177_00302228231218562.pdf]

Supplementary material

Table 1. Preoccupation with cognitions when it was as its worst (n=8).

	**Preoccupation with Mental imagery**	**Preoccupation with Verbal thoughts**
Nr of participants with the experience ***all the time***	2	0
Nr of participants with the experience ***half of the time or more***	7	5
Median score, scale 1-9 (range)	5 (2 - 9)	5 (1 - 8)

Table 2. Preoccupation with suicide-related cognitions when it was as its worst (n=8).

	**Preoccupation with Suicide-related Mental imagery**	**Preoccupation with Suicide-related Verbal thoughts**
Nr of participants with the experience ***all the time***	4	3
Nr of participants with the experience ***half of the time or more***	7	5
Median score, scale 1-9 (range)	8 (3 - 9)	6,5 (1 - 9)

Table 3. Experienced reality of cognitions when it was as its worst (n=8).

	**Experienced reality of Mental imagery**	**Experienced reality of Verbal thoughts**
Nr of participants with the experience, ***as if it was real***	7	6
Nr of participants with the experience, ***half real or more***	8	7
Median score, scale 1-9 (range)	9 (7 - 9)	9 (1 - 9)

Table 4. How compelling the cognitions were experienced when it was as its worst (n=8).

	**How compelling the Mental imagery was experienced**	**How compelling the Verbal thoughts were experienced**
Nr of participants with the experience, ***entirely compelling***	4	3
Nr of participants with the experience, ***moderately compelling or more***	6	4
Median score, scale 1-9 (range)	7 (1 - 9)	6,5 (1 - 9)

Table 5. Number of participants that experienced the respective item in checklist (n=8).

**Category in the checklist**	**Mental imagery**	**Verbal thoughts**
Of a distressful event that you experienced (trauma)	4	4
Of a previous occasion when you tried to harm yourself	1	3
Of yourself when you plan/prepare to harm yourself or attempt suicide in the future	5	5
Of things that you escaped from	7	6
Of what might happen *to you* if you died	4	5
Of what might happen *to others* if you died	5	4
That made you feel more secure and better	3	5
That were volatile or unclear	3	4
Any other type	1	4
